# Comparison of Surgical Site Infections in Laparoscopic Versus Open Appendectomy: A Prospective Cohort Study

**DOI:** 10.7759/cureus.80530

**Published:** 2025-03-13

**Authors:** Arsalan Shah Roghani, Farhan Shahzad, Faizan Shah Roghani, Munayal Roghani, Zaryab Khan, Asim Ullah, Sanan Khan, Yasir Mahmood

**Affiliations:** 1 Surgery, Khyber Teaching Hospital, Peshawar, PAK; 2 Internal Medicine, Saidu Group of Teaching Hospitals, Swat, PAK; 3 General Surgery, Hayatabad Medical Complex Peshawar, Peshawar, PAK; 4 Suregry, Khyber Teaching Hospital, Peshawar, PAK

**Keywords:** acute appendicitis, cohort study, laparoscopic appendectomy, open appendectomy, surgical site infection

## Abstract

Aim

To compare the incidence of surgical site infections (SSIs) in laparoscopic appendectomy vs. open appendectomy.

Methodology

A total of 80 patients with an acute appendicitis diagnosis were chosen and split into two groups: Group A (laparoscopic appendectomy) and Group B (open appendectomy), and permission from the hospital's ethical committee was obtained before patient enrollment. Eighty patients, of either gender, undergoing laparoscopic and open appendectomy were enrolled in a prospective cohort study. On the first, second, third, fifth, and seventh days following surgery, the wound was evaluated and scored in accordance with the wound asepsis score. Then, the overall wound asepsis score and SSIs were computed. In both groups, SSIs were compared. IBM SPSS Statistics for Windows, Version 23 (Released 2015; IBM Corp., Armonk, NY, USA) was used to analyze the results by applying the Chi-square test and Student t-test, which were then presented in the form of descriptions, statistical tables, and charts.

Results

Group A's mean age was 41.70 ± 12.195 years, whereas Group B's mean age was 43.75 ± 9.131 years (p = 0.39). Group A's and Group B's BMIs were 24.65 ± 2.98 and 25.90 ± 3.07 (p = 0.06), respectively, and the male-to-female ratios were 1.2:1 and 1.6:1, respectively. SSIs were notably higher in the open appendectomy group (12, or 30%) compared to the laparoscopic group (4, or 10%) (p = 0.02).

Conclusion

The incidence of SSIs was notably higher in the open appendectomy group as compared to the laparoscopic appendectomy group; therefore, laparoscopic appendectomy is a safer approach toward acute appendicitis, as compared to open appendectomy in terms of SSIs.

## Introduction

Infections acquired through the hospital, especially surgical site infections (SSIs), significantly increase morbidity, lengthen hospital stays, and impede the healing of wounds. After surgery, SSIs can occur within 30 days (or a year for implants) and can be superficial, deep, or organ-space. Over 50% of SSIs are caused by *Staphylococcus aureus*, including methicillin-resistant strains. This prevalent culprit is responsible for the majority of SSIs. Other skin-derived microbes can also lead to infections, which can affect wounds as well as implanted prostheses. Appendectomy is a common abdominal surgery that is often required due to acute appendicitis, which affects 7%-12% of the population [1]. SSIs pose a substantial risk in the healthcare industry, especially following surgical interventions such as an appendectomy. An appendectomy is a surgical procedure that involves the removal of the appendix [2]. It is a frequently performed emergency surgery on a global scale. Although the occurrence of SSIs has decreased due to improvements in surgical methods and infection control measures, they nevertheless provide a potential danger to patients undergoing this treatment [3]. SSIs may arise as a result of multiple factors, encompassing the patient's overall health condition, the specific surgical setting, and the nature of the surgical intervention [4]. The development of SSIs following an appendectomy is influenced to a considerable extent by patient-related factors. Individuals who have pre-existing medical disorders, such as diabetes, obesity, or immunodeficiency, have an elevated susceptibility to adverse outcomes as a result of poor wound healing and compromised immune response [5].

Extensive evidence has unequivocally established the superiority of laparoscopic surgery over open surgery [6]. The occurrence of SSIs, the formation of incisional hernias, and wound dehiscence have been found to be associated with an open appendectomy [6]. Open appendectomy is the conventional treatment for perforated appendicitis, which involves the removal of the appendix through an open incision, and there is insufficient data regarding the utilization of laparoscopic appendectomy, where the appendix is removed through a trocar [7]. Before that, open appendectomies were performed using the open approach, and the first laparoscopic appendectomy was done in 1983 [1].

Laparoscopic appendectomies are conducted via a small surgical cut made in the abdominal region of the patient [7]. Research has demonstrated that laparoscopic appendectomy offers notable benefits over open appendix surgery. These benefits include decreased discomfort following surgery, accelerated wound healing, and a lower incidence of wound infections; however, one notable advantage of laparoscopic appendectomy, in comparison to open appendectomy, is the ability to directly observe the peritoneum throughout the rinsing process, which serves to mitigate the risk of peritonitis. Laparoscopic appendectomy is favored over open appendectomy due to this primary factor. Furthermore, a significant association has been observed between laparoscopic appendectomy and a reduced occurrence of wound contamination [8-11].

Infection of wounds after surgery is a prevalent issue in healthcare and can lead to considerable morbidity, death, and resource consumption [1]. The wound infection process is intricate and entails the interaction of multiple biological processes at the molecular level. Wound infections are associated with significant rates of morbidity and mortality. Due to the scarcity of literature on this subject locally, the goal of this study is to compare laparoscopic vs. open appendectomy in terms of SSIs. Laparoscopic surgery has the potential to provide several benefits in terms of reducing rates of SSIs. These advantages include smaller incisions, reduced tissue trauma, and faster recovery. However, it is important to consider specific patient characteristics and surgical considerations when selecting a surgical approach. This approach can help improve outcomes and minimize complications, such as SSIs.

## Materials and methods

We conducted this prospective cohort study at the Department of Surgery of Khyber Teaching Hospital, Peshawar, Pakistan, from January 2023 to June 2023, after obtaining ethical approval from the hospital's ethical committee. The sample size chosen for this study was 80 patients, divided into two groups (block randomization): 40 patients in Group A (laparoscopic appendectomy) and 40 patients in Group B (open appendectomy), by applying a 95% confidence interval and 80% power of the test. The diagnosis of acute appendicitis was made using the patient's physical examination, medical history, blood counts, and abdominal/pelvic ultrasonography. Patients were aged 18 to 60 years of either gender. Postoperative pain, operative time, hospital stay, and SSIs were noted as outcomes in both groups. Patients who had an Alvarado score of >5 were included in the study. Individuals who did not provide consent to be included in the study, could not be clinically diagnosed with appendicitis, or who had contraindications for laparoscopy were excluded from the study. They were informed of the study's objectives and purpose and given the assurance that it was being conducted purely for research. Informed consent was acquired if they consented. General anesthesia was administered to every patient, and preoperative antiseptic procedures (preoperatively, the surgical area was shaved, and prophylactic antibiotics were administered to all patients) were the same for both patient groups. A skilled surgeon with over five years of expertise carried out the surgeries, and as per the preoperative antibiotic regimen, a 1-gram injection of Cebac (cefoperazone and sulbactam) was used.

An open appendectomy was performed in the right iliac fossa, resulting in the splitting of the abdominal muscles and the ligation and removal of the appendix from its base. With caution, any potentially pathogenic material was kept from spilling during the removal of the appendix. Three ports were placed onto the abdominal wall during a laparoscopic appendectomy: two 5-mm ports and one 10-mm port. This was done after the pneumoperitoneum was created. The appendix was knotted at the base, mobilized, and then removed through one of the ports to be withdrawn to the trocar, and factors such as mean operative time were recorded. All the patients were given an injection of 1-gram Cebac (cefoperazone and sulbactam), twice daily (morning and evening), in the postoperative period. The patients who were experiencing pain were controlled with injection Toradol (ketorolac) as needed. The wound was assessed and evaluated on the first, second, third, fifth, and seventh postoperative days and was documented if present. After calculating the overall wound asepsis score, SSIs were classified as follows: no wound infection for a total score between 0 and 20, and SSIs for a total score greater than 20 (score of 0-10 for wound healed satisfactorily, 11-20 for disturbance of healing, 21-30 for minor wound infection, 31-40 for moderate wound infection, and a score of 40 or above for severe wound infections). Patients were discharged with a five-day supply of Calamox tablets (amoxicillin and clavulanic acid), twice daily (morning and evening), after receiving appropriate counseling regarding avoiding physical exercise and regular wound care. Chi-square and t-tests were used for comparison, keeping the p-value significant at ≤0.05.

IBM SPSS Statistics for Windows, Version 23 (Released 2015; IBM Corp., Armonk, NY, USA), was the statistical program used to analyze the data. Age and mean operative time were examples of continuous variables that were calculated as means ± standard deviation. The proportions of several categorical factors, including gender and SSIs, were examined. The study employed stratification based on age, gender, and appendectomy procedure in both groups. To account for confounders and assumptions, Chi-square and the Student's t-test were utilized. A p-value of less than 0.05 was deemed significant. Tables and graphs were used to display each result.

## Results

A total of 80 acute appendicitis patients were selected. In Group A (laparoscopic appendectomy), the mean age was 41.70 ± 12.195 years, and in Group B (open appendectomy), the mean age was 43.75 ± 9.131 years (p = 0.39). The BMI was 24.65 ± 2.98 in Group A and 25.90 ± 3.07 in Group B (p = 0.06) (Table 1).

**Table 1:** Demographics Data are presented in the table, and p-values were calculated using the Chi-square test.

Demographics	Groups	N	Mean	Std. deviation	p-value
Age (years)	Group A (laparoscopic appendectomy)	40	41.70	12.195	0.39
	Group B (open appendectomy)	40	43.75	9.131	
BMI (kg/m^2^)	Group A (laparoscopic appendectomy)	40	24.65	2.98	0.06
	Group B (open appendectomy)	40	25.90	3.07	

**Figure 1:**
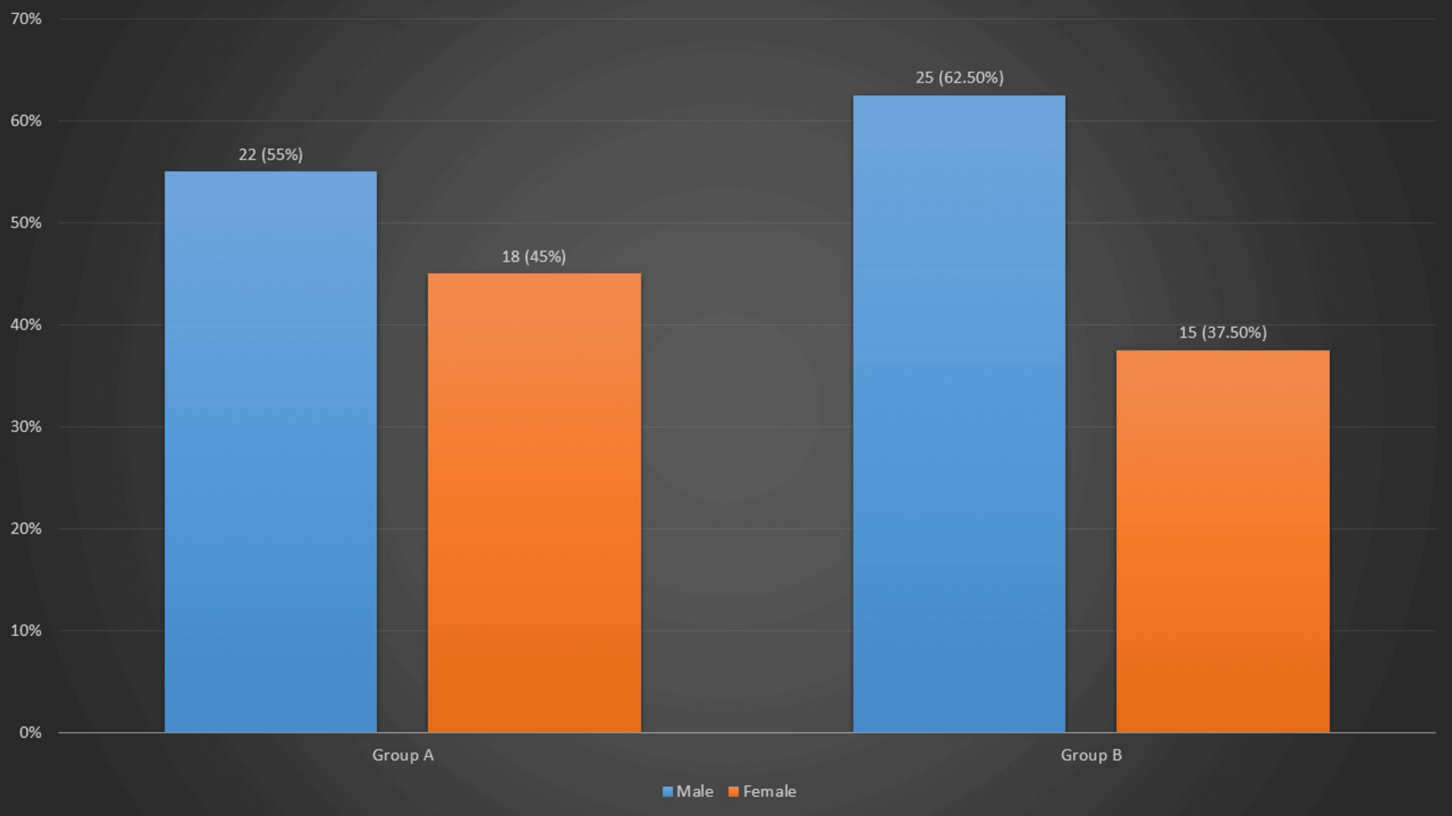
Gender distribution

The gender-wise distribution revealed that the frequency of male patients in Group A was 22 (55%), while the frequency of female patients was 18 (45%). In Group B, the frequency of male patients was 25 (62.5%), while the frequency of female patients was 15 (37.5%) (Figure 1).

In Group A, the operative time was notably longer than in Group B (p = 0.0001), while the hospital stay was notably shorter in Group A (p = 0.0001) (Table 2). The pain score on the visual analog scale (VAS) between both groups showed no notable difference; however, 16 (40%) patients had mild pain in Group A, and 11 (27.5%) patients had mild pain in Group B. Moderate pain was almost similar in both groups, while Group B exhibited a higher frequency of severe pain, with 15 (37.5%) patients experiencing it (Table 3).

**Table 2:** Comparison of operative time and hospital stay between both groups Data are presented in the table, and p-values were calculated using the Chi-square test.

Groups		N	Mean	Std. deviation	p-value
Operative time (mins)	Group A (laparoscopic appendectomy)	40	48.03	7.781	0.0001
	Group B (open appendectomy)	40	35.83	6.046	
Hospital stay (days)	Group A (laparoscopic appendectomy)	40	1.90	0.744	0.0001
	Group B (open appendectomy)	40	4.13	1.223	

**Table 3:** Comparison of pain score between both groups Data are presented in the table, and p-values were calculated using the Student's t-test. VAS: visual analog scale

		Pain (VAS)			Total	p-value
		Mild	Moderate	Severe		
Groups	Group A (laparoscopic appendectomy)	16	15	9	40	0.29
		40.0%	37.5%	22.5%	100.0%	
	Group B (open appendectomy)	11	14	15	40	
		27.5%	35.0%	37.5%	100.0%	
Total		27	29	24	80	
		33.8%	36.2%	30.0%	100.0%	

We observed that the incidence of SSIs was notably higher in the open appendectomy group (12 or 30%) as compared to the laparoscopic group (4 or 10%) (p = 0.02) (Table 4).

**Table 4:** Comparison of surgical site infection between both groups Data are presented in the table, and p-values were calculated using the Student's t-test.

		Surgical site infection		Total	p-value
		Yes	No		
Groups	Group A (laparoscopic appendectomy)	4	36	40	0.02
		10.0%	90.0%	100.0%	
	Group B (open appendectomy)	12	28	40	
		30.0%	70.0%	100.0%	
Total		16	64	80	
		20.0%	80.0%	100.0%	

## Discussion

One of the most common surgical procedures carried out all over the world is called an appendectomy, which includes the removal of the appendix using surgical means. When it comes to treating acute appendicitis, the conventional method of treatment has traditionally been an open appendectomy [12]. In spite of this, laparoscopic appendectomy has become increasingly popular as a minimally invasive alternative because of the breakthroughs that have been made in surgical procedures. When deciding between laparoscopic and open appendectomy, the decision is influenced by a number of factors, such as the state of the patient, the expertise of the surgeon, and the resources available at the hospital [13-16]. For many years, appendectomies have been performed using the traditional open approach. According to studies, 33% of women who are not pregnant but of reproductive age receive an inaccurate diagnosis of appendicitis; yet, laparoscopic surgery is now commonly considered to be preferable in numerous aspects. Laparoscopic appendectomy is better than open appendectomy because the entire peritoneal cavity may be visualized. Misdiagnosis of gynecological problems and female functioning abnormalities is common in women. Thus, laparoscopic appendectomy helps prevent unnecessary appendectomy and improves diagnostic accuracy in patients with suspected appendicitis [1]. The procedure, known as laparoscopic appendectomy, requires the patient to make a number of small incisions in the belly in order to introduce a camera and specialized instruments for the removal of the appendix. Open appendectomy, on the other hand, necessitates a more extensive incision in the abdominal wall in order to directly reach and remove the appendix that is not functioning properly [13-16]. When it comes to the treatment of acute appendicitis, only a few studies have been carried out in poor countries that compare the two different modalities utilized [17,18].

We conducted our study on 80 patients undergoing appendectomy; Group A patients had laparoscopic appendectomy, while Group B patients had open appendectomy. Demographic comparison showed no noticeable difference, including age, gender, and BMI; however, we observed a higher frequency of male patients in our study in both groups.

Operative time in the laparoscopic group was notably higher when compared with the open appendectomy group. This observation has been reported in several studies. A study conducted in Pakistan showed that operative time was higher in laparoscopic appendectomy [19].

Hospital stay was notably higher for the open appendectomy group when compared to the laparoscopic group. In contrast to our observations, the aforementioned studies conducted in Pakistan also reported that the difference in hospital stay between both groups was not significant [19].

The primary measure of outcome in our study was to assess SSIs in both groups. Group A showed a significantly lower incidence of SSIs than Group B; around 10% of patients in Group A had SSIs, while 30% of patients in Group B had SSIs (p = 0.02). Comparable results were obtained from a meta-analysis of 2,877 participants who were enrolled in 28 trials. Although the total risk of complications was similar, wound infections significantly decreased (2.3% from 6.1%) after laparoscopy [1]. A local study reported similar observations; they conducted a similar study and observed that the laparoscopic group had a 10.77% incidence of SSIs, while the open appendectomy group had a 27.69% incidence of SSIs [20]. A similar observation was reported by the aforementioned local study. They graded their patients on the Southampton Grading system for SSIs and found that the incidence of SSIs was higher in the open appendectomy group [19].

Limitations

The following are the limitations of the study: (1) small sample size, (2) single-center study (conducted in one hospital), (3) no blinding (the study did not use blinding to reduce bias), and (4) limited outcome measures. The study primarily focused on SSIs, hospital stay, and pain scores but did not examine other important outcomes, such as long-term quality of life or cost-effectiveness.

## Conclusions

According to the results of this study and the findings from international literature on this topic, we can safely conclude that laparoscopic appendectomy is superior to open appendectomy in terms of lower incidence of SSIs, hospital stay, and pain on the VAS scale. It also offers the benefit of diagnostic laparoscopy in cases where the diagnosis is uncertain. Open appendectomy exhibited a shorter operative time than laparoscopic appendectomy.
